# Comparison of dabigatran, rivaroxaban, and apixaban for effectiveness and safety in atrial fibrillation: a nationwide cohort study

**DOI:** 10.1093/ehjcvp/pvz086

**Published:** 2020-01-14

**Authors:** Ole-Christian W Rutherford, Christian Jonasson, Waleed Ghanima, Fabian Söderdahl, Sigrun Halvorsen

**Affiliations:** 1 Department of Cardiology, Østfold Hospital Trust, PO Box 300, 1714 Grålum, Sarpsborg, Norway; 2 Institute of Clinical Medicine, University of Oslo, PO Box 1171 Blindern, 0318 Oslo, Norway; 3 HUNT Research Centre, Faculty of Medicine and Health Sciences, NTNU—Norwegian University of Science and Technology, Forskningsveien 2, 7600 Levanger, Norway; 4 Department of Haematology, Østfold Hospital Trust, PO Box 300, 1714 Grålum. Sarpsborg, Norway; 5 Statisticon AB, Klara Södra kyrkogata 1, 111 52 Stockholm, Sweden; 6 Department of Cardiology, Oslo University Hospital Ullevål, PO Box 4956, Nydalen. NO-0424 Oslo, Norway

**Keywords:** Atrial fibrillation, Non-vitamin K antagonist anticoagulants, Stroke, Bleeding

## Abstract

**Aims:**

The aim of this study was to compare the risk of stroke or systemic embolism (SE) and major bleeding in patients with atrial fibrillation (AF) using dabigatran, rivaroxaban, and apixaban in routine clinical practice.

**Methods and results:**

Using nationwide registries in Norway from January 2013 to December 2017, we established a cohort of 52 476 new users of non-vitamin K antagonist oral anticoagulants (NOACs) with AF. Users of individual NOACs were matched 1:1 on the propensity score to create three pairwise-matched cohorts: dabigatran vs. rivaroxaban (20 504 patients), dabigatran vs. apixaban (20 826 patients), and rivaroxaban vs. apixaban (27 398 patients). Hazard ratios (HRs) for the risk of stroke or SE and major bleeding were estimated. In the propensity-matched comparisons of the risk of stroke or SE, the HRs were 0.88 [95% confidence interval (CI) 0.76–1.02] for dabigatran vs. rivaroxaban, 0.88 (95% CI 0.75–1.02) for dabigatran vs. apixaban, and 1.00 (95% CI 0.89–1.14) for apixaban vs. rivaroxaban. For the risk of major bleeding, the HRs were 0.75 (95% CI 0.64–0.88) for dabigatran vs. rivaroxaban, 1.03 (95% CI 0.85–1.24) for dabigatran vs. apixaban, and 0.79 (95% CI 0.68–0.91) for apixaban vs. rivaroxaban.

**Conclusion:**

In this nationwide study of patients with AF in Norway, we found no statistically significant differences in risk of stroke or SE in propensity-matched comparisons between dabigatran, rivaroxaban, and apixaban. However, dabigatran and apixaban were both associated with significantly lower risk of major bleeding compared with rivaroxaban.

## Introduction

Oral anticoagulants (OACs) are effective in preventing stroke and systemic embolism (SE) in patients with atrial fibrillation (AF) but are associated with an increased risk of bleeding.^1^ Guidelines recommend use of non-vitamin K antagonist oral anticoagulants (NOACs) over traditional therapy with vitamin K antagonists in most patients,^2^ and the number of patients being treated with NOACs has increased rapidly during the last few years.^3^ In the pivotal randomized controlled trials (RCTs) leading to their approval, each NOAC was compared with warfarin,^4–6^ however, no head-to-head comparison between the individual NOACs has been performed. In the absence of RCTs, observational studies utilizing data from clinical practice may add useful information regarding comparative effectiveness and safety of the individual NOACs. The aim of this study was to assess the association between the use of dabigatran, rivaroxaban, and apixaban and the risk of stroke or SE and bleeding in a nationwide cohort of patients with AF.

## Methods

### Data sources

The Norwegian Patient Registry (NPR) is a nationwide registry that covers all hospital admissions and outpatient consultations as well as all specialist consultations in Norway. Each admission or consultation is assigned a primary (the disease or condition being treated) and secondary cause (relevant comorbidities). Diagnoses are coded according to the International Classification of Diseases, 10th revision (ICD-10)^7^ system and surgical procedures are coded according to the Nordic Medico-Statistical Committee (NOMESCO) coding system.^8^^,^^9^

The Norwegian Prescription Database (NorPD) holds information on all drug prescriptions dispensed from pharmacies nationwide. Drugs are coded according to the Anatomical Therapeutic Chemical (ATC) system.^10^ The Norwegian system of general reimbursement of medicine expenses requires the prescribing physician to state the relevant underlying disease warranting each drug’s reimbursement. The NorPD also contains information about date of dispensation, quantity, and strength of drugs dispensed.

### Cohort creation and study design

**Figure 1:**
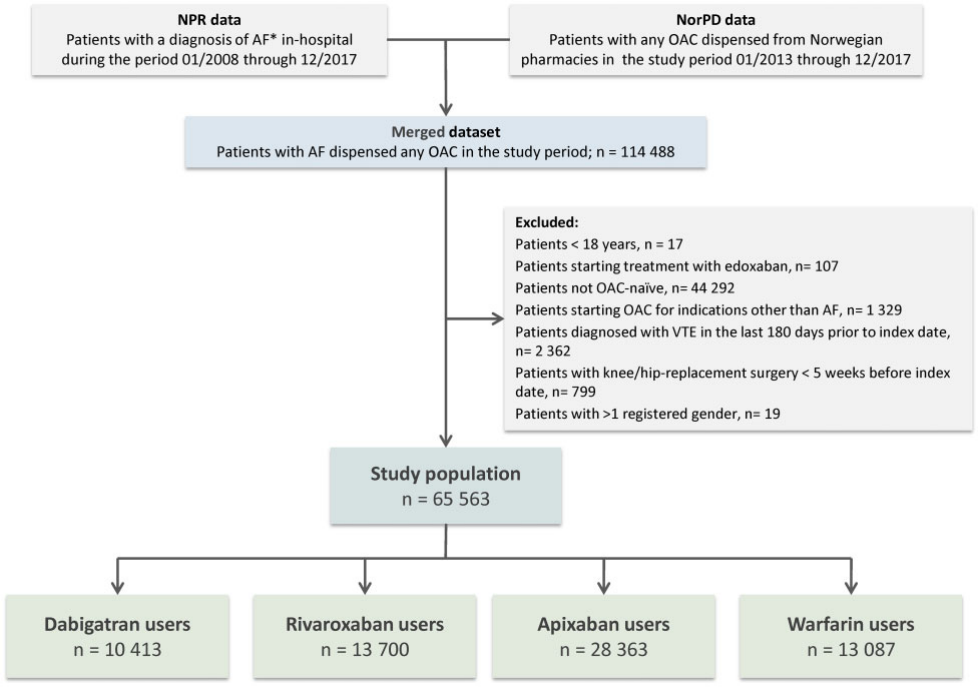
Cohort creation flow chart. AF*, atrial fibrillation in the absence of mitral stenosis or mechanical prosthetic heart valves; NPR, Norwegian Patient Registry; NorPD, Norwegian Prescription Database; OAC, oral anticoagulant; VTE, venous thromboembolism.

The study cohort was generated by linkage of data from the NPR and the NorPD (*Figure 1*). The study population included all patients **≥**18 years diagnosed with AF with at least one OAC dispensation (dabigatran 110 mg or 150 mg, rivaroxaban 15 mg or 20 mg, apixaban 2.5 mg or 5 mg, or warfarin 2.5 mg) in the study period (January 2013 to December 2017) but being anticoagulant naïve before start of the study. Patients initiating warfarin were included to enable comparisons between our findings and previous studies including patients treated with warfarin. Patients were excluded if they had mitral stenosis or mechanical prosthetic heart valves. Anticoagulant-naïve was defined as no dispensing of anticoagulants from pharmacies in the preceding 12 months before the index date. The index date was defined as the date of the first dispensation of an OAC in the study period. Due to limited usage in the study period, patients initiating edoxaban were excluded (*n* = 107). Patients with a history of venous thromboembolism during the last 180 days, or knee- or hip replacement surgery during the last 35 days before the index date were excluded. Details of the cohort creation procedure are shown in *Figure 1*, and ICD-10 codes used for inclusion- and exclusion criteria are listed in the Supplementary material online, *Table S1*.

Patients treated with a NOAC were matched with respect to propensity score, and three pairwise-matched cohorts were created: dabigatran vs. rivaroxaban; dabigatran vs. apixaban; and apixaban vs. rivaroxaban. Details of the propensity score matching (PSM) are found in the section on statistical analysis.

### Comorbidities

Diagnoses for all hospital admissions, consultations, and procedures in the previous 5 years before the index date were retrieved from the NPR. A medication history of 5 years, including all relevant diagnosis-specific reimbursement codes, was completed from the NorPD. This information was used to compile a set of comorbidities and medication history for each patient, using primary as well as secondary codes related to each admission. The ICD-10 codes included for each diagnosis are shown in Supplementary material online, *Table S1*, and Supplementary material online, *Table S2* shows in detail how CHA_2_DS_2_-VASc- and HAS-BLED scores were calculated.

### Oral anticoagulant supply

For each OAC, the days of supply were computed using information on dates of dispensing, the pack-size dispensed, and the number of packages. As the NOACs are prescribed in fixed doses, to be taken once daily (rivaroxaban) or twice daily (dabigatran and apixaban), the number of days of supply strictly corresponds to the amount dispensed. The days of warfarin supply were estimated as previously described.^11^ To account for incomplete adherence, a 30-day gap period between the calculated end of OAC supply and the date of a new prescription was allowed, before patients were censored.

### Outcomes and follow-up

Outcome measures of effectiveness were time to first stroke (haemorrhagic or ischaemic) or SE, and time to first ischaemic stroke. Outcome measures of safety were time to first major bleeding, clinically relevant non-major bleeding (CRNM bleeding), major or CRNM bleeding, gastrointestinal bleeding (GI bleeding), and intracranial haemorrhage. Major bleeding was defined as previously described as any bleeding into a critical area or organ, or any bleeding accompanied by blood transfusion ≤10 days after hospital admission date.^11^ CRNM bleeding was defined according to the International Society on Thrombosis and Haemostasis (ISTH) classification,^12^ as any bleeding necessitating intervention by a medical professional. ICD-10 and NOMESCO codes used for identification of outcomes are listed in Supplementary material online, *Table S1*. Patients were followed from the index date until discontinuation or switching of OACs, death, or end of study period (31 December 2017), whichever occurred first. For the identification of effectiveness- and safety outcomes, only primary (first listed) ICD-10 codes for each hospital stay were used.

### Ethics

Registration of data into the NPR and the NorPD is mandatory in Norway and legally exempt from obtainment of patient consent. This study was approved by the Regional Ethical Committee (Ref. No. 2017/410/REK North).

### Statistical analysis

Categorical variables are reported by numbers and percent, continuous variables by means with standard deviations. Cox proportional hazards regression was used to select the strongest predictor variables for stroke/SE and major bleeding. The proportional hazards assumption was checked using Schoenfeld residuals, and by comparing the log–log transformation of the Kaplan–Meier survival curves for each variable.^13^

To account for confounding by indication of therapy, PSM was performed. Using logistic regression, the probability of a patient being prescribed a specific NOAC was calculated on the basis of the following 16 covariates; age, gender, chronic kidney disease, hypertension, diabetes, ischaemic heart disease, peripheral artery disease, heart failure, history of stroke/SE, history of bleeding-related hospitalization, anaemia, active cancer (cancer diagnosis last 12 months), chronic lower respiratory tract disease, use of cholesterol lowering drugs, use of antiplatelet drugs, and use of non-steroidal anti-inflammatory drugs during the last 12 months. For each patient initiating a specific NOAC, initiators of another NOAC to be compared were matched 1:1 on the logit of the propensity score using calipers of width equal to 0.2 of the standard deviation of the logit of the propensity score.^14^ Three propensity score-matched sets were constructed; dabigatran matched with rivaroxaban, dabigatran matched with apixaban, and rivaroxaban matched with apixaban. The balance between treatment populations was assessed by investigating absolute standardized mean differences of all baseline covariates before and after the matching, using a threshold of 0.1 to indicate imbalance. Cox regression with robust sandwich estimates was utilized for evaluating the rates of stroke and bleeding in the propensity score-matched groups.^15^ As the matched sets were balanced, NOAC treatment was entered as the only independent variable.^16^^,^^17^ Subgroup analyses were performed investigating the risk of stroke and major bleeding in specific subgroups; age (<75 years vs. >75 years), gender, history of stroke, and history of bleeding. For the analyses stratified on the initial dose, *de novo* PSM within the initial dose defined subgroups were performed. Adjusted hazard ratios (HRs) along with *P*-values for interaction between treatment and the specific subgroup were calculated.

Three sensitivity analyses were performed: (i) the analyses of the outcomes stroke/SE and major bleeding in the PSM cohorts were repeated restricting the follow-up time for all NOACs to 12 months; (ii) an ‘intention-to-treat’-like analysis: the analyses of the outcomes stroke/SE and major bleeding in the PSM cohorts were performed without censoring by treatment switch or discontinuation of NOACs. (iii) The comparative analyses of the outcomes stroke/SE and major bleeding were repeated in the full dataset using conventional adjustment instead of PSM to avoid exclusion of non-matched patients from the analyses.

Finally, as a *post hoc* analysis, we performed NOAC–warfarin comparisons. The risk of stroke/SE and major bleeding were compared between users of dabigatran, rivaroxaban, apixaban, and users of warfarin, using a Cox proportional hazards model with conventional adjustment.

Level of significance was set to 5%. We did not adjust for multiple comparisons. Statistical analyses were performed using SAS v.9.4 (SAS Institute, Inc.) and STATA v.15 (STATACorp LLC).

## Results

A total of 65 563 new users of OACs were identified and included in the study population; 10 413 initiated dabigatran, 13 700 rivaroxaban, 28 363 apixaban, and 13 087 initiated warfarin (*Figure 1*). Baseline characteristics for the unmatched groups are shown in Supplementary material online, *Table S3*. New users of dabigatran were more likely to be younger than new users of the other drugs, and they also had less comorbidity. The mean CHA_2_DS_2_-VASc- and HAS-BLED scores were lowest in users of dabigatran. The standard dose for stroke prevention was used in 63.9% of dabigatran patients, 75.6% of rivaroxaban patients, and 74.6% of apixaban patients.

### Non-vitamin K antagonist oral anticoagulant–non-vitamin K antagonist oral anticoagulant comparisons

**Figure 2:**
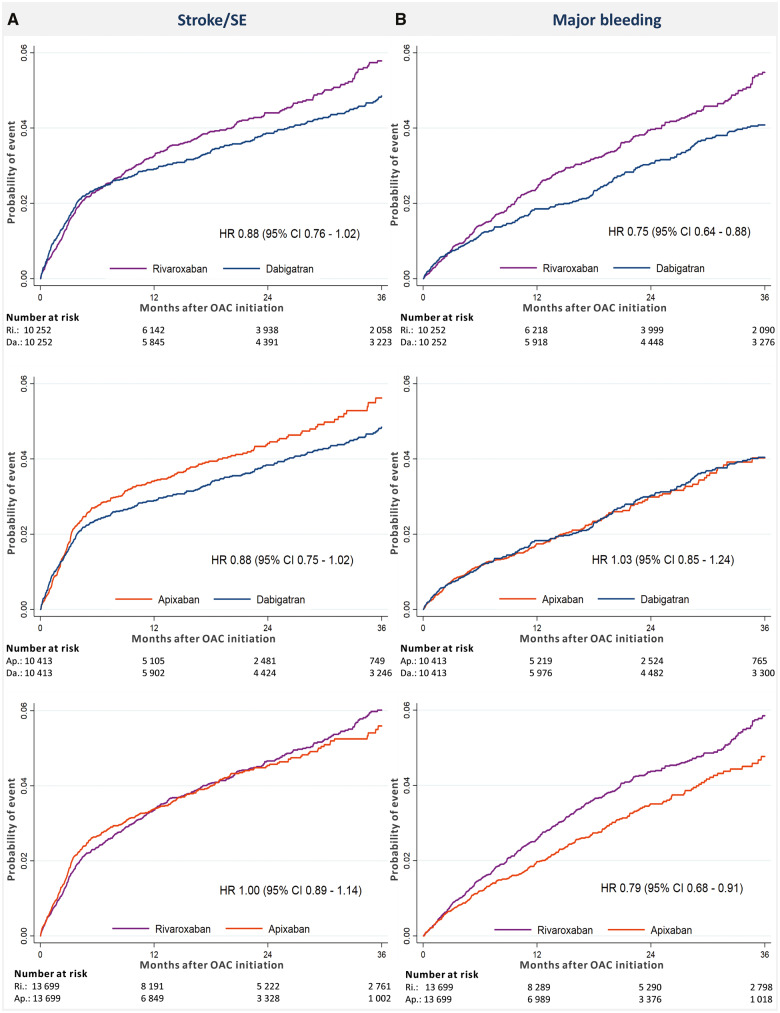
Cumulative incidence of stroke or systemic embolism (*A*) and major bleeding (*B*) in the propensity score-matched groups. CI, confidence interval; HR, hazard ratio; OAC, oral anticoagulant; SE, systemic embolism.

**Figure 3:**
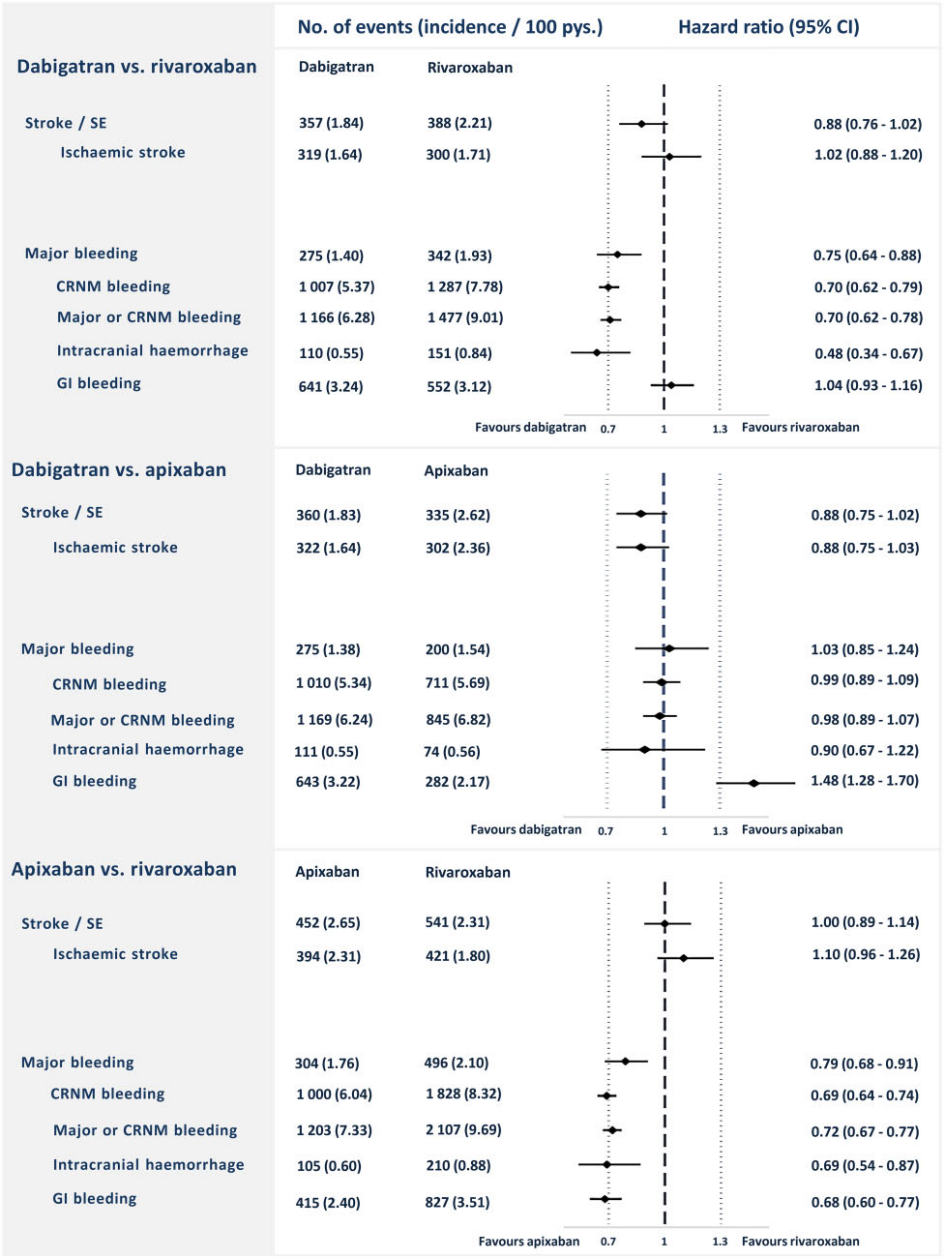
Number of events, incidence rates, and hazard ratios for primary and secondary outcomes in the three propensity score-matched cohorts. CI, confidence interval; CRNM, clinically relevant non-major; GI, gastrointestinal; Pys., person-years; SE, systemic embolism.

After PSM in a 1:1 ratio, the cohorts used in the analyses of dabigatran vs. rivaroxaban included a total of 20 504 patients, dabigatran vs. apixaban included a total of 20 826 patients, and rivaroxaban vs. apixaban included a total of 27 398 patients. In each of the matched cohorts, baseline characteristics were well-balanced between the groups (*Table 1*). Plots of propensity scores before and after matching are shown in the Supplementary material, *Figure S1*. *Figure 2* shows Kaplan–Meier curves for the risk of stroke/SE and major bleeding, whereas *Figure 3* shows the incidence rates and HRs of the outcomes stroke/SE and major bleeding for the three PSM cohorts. The proportional hazard assumption was fulfilled for all primary analyses.

**Table 1:** Baseline characteristics, propensity-matched groups

	Dabigatran–rivaroxaban-matched cohort (*n* = 20 504)			Dabigatran–apixaban-matched cohort (*n* = 20 826)			Apixaban–rivaroxaban-matched cohort (*n* = 27 398)		
	Dabigatran (*n* = 10 252)	Rivaroxaban (*n* = 10 252)	SMD	Dabigatran (*n* = 10 413)	Apixaban (*n* = 10 413)	SMD	Apixaban (*n* = 13 699)	Rivaroxaban (*n* = 13 699)	SMD
Age									
Mean (SD)	70.9 (10.95)	70.9 (11.21)	0.004	70.6 (11.18)	70.6 (11.67)	<0.001	72.7 (11.66)	72.7 (11.08)	<0.001
Median	71	71		71	71		73	73	
<65 years	2526 (24.6)	2614 (25.5)		2687 (25.8)	2794 (26.8)		2934 (21.4)	2786 (20.3)	
65–74 years	3869 (37.7)	3748 (36.6)		3869 (37.2)	3759 (36.1)		4596 (33.5)	4805 (35.1)	
≥75 years	3857 (37.6)	3890 (37.9)		3857 (37.0)	3860 (37.1)		6169 (45.0)	6108 (44.6)	
OAC dose									
Standard dose	6498 (63.4)	8115 (79.2)		6652 (63.9)	8514 (81.8)		10 508 (76.7)	10 362 (75.6)	
Reduced dose	3754 (36.6)	2137 (20.8)		3761 (36.1)	1899 (18.2)		3191 (23.3)	3337 (24.4)	
Male gender	6286 (61.3)	6313 (61.6)	0.005	6433 (61.8)	6447 (61.9)	0.003	7946 (58.0)	7943 (58.0)	0.000
Hypertension	6656 (64.9)	6628 (64.7)	0.006	6693 (64.3)	6641 (63.8)	0.010	9376 (68.4)	9288 (67.8)	0.014
Ischaemic heart disease	2107 (20.6)	2089 (20.4)	0.004	2119 (20.3)	2101 (20.2)	0.004	3050 (22.3)	3061 (22.3)	0.002
Vascular disease	743 (7.2)	757 (7.4)	0.005	743 (7.1)	720 (6.9)	0.009	1265 (9.2)	1262 (9.2)	0.001
Heart failure	2103 (20.5)	2100 (20.5)	0.001	2140 (20.6)	2079 (20.0)	0.015	3029 (22.1)	3043 (22.2)	0.002
Chronic kidney disease	245 (2.4)	258 (2.5)	0.008	245 (2.4)	255 (2.4)	0.006	657 (4.8)	627 (4.6)	0.010
Diabetes mellitus	1318 (13.2)	1352 (13.2)	0.010	1324 (12.7)	1294 (12.4)	0.009	1923 (14.0)	1887 (13.8)	0.008
Chronic lower respiratory tract diseases	1137 (11.1)	1122 (10.9)	0.005	1141 (11.0)	1128 (10.8)	0.004	1654 (12.1)	1632 (11.9)	0.005
Active cancer (diagnosis last 12 months)	769 (7.5)	770 (7.5)	0.000	770 (7.4)	773 (7.4)	0.001	1276 (9.3)	1263 (9.2)	0.003
History of stroke/SE	1341 (13.1)	1330 (13.0)	0.003	1356 (13.0)	1322 (12.7)	0.010	1860 (13.6)	1792 (13.1)	0.015
History of anaemia	456 (4.4)	447 (4.4)	0.004	458 (4.4)	432 (4.1)	0.012	801 (5.8)	757 (5.5)	0.014
History of bleeding	1142 (11.1)	1142 (11.1)	0.000	1144 (11.0)	1097 (10.5)	0.015	1723 (12.6)	1715 (12.5)	0.002
Use of antiplatelet drugs last 12 months	5109 (49.8)	5079 (49.5)	0.006	5125 (49.2)	5016 (48.2)	0.021	7312 (53.4)	7207 (52.6)	0.015
Use of NSAIDs last 12 months	2485 (24.2)	2467 (24.1)	0.004	2512 (24.1)	2492 (23.9)	0.004	3047 (22.2)	3148 (23.0)	0.018
Use of cholesterol lowering drugs	4603 (44.9)	4598 (44.8)	0.001	4629 (44.5)	4516 (43.4)	0.022	6356 (46.4)	6315 (46.1)	0.006
Mean CHA_2_DS_2_-VASc score (SD)	2.99 (1.73)	2.98 (1.71)	0.006	2.96 (1.74)	2.93 (1.72)	0.017	3.23 (1.74)	3.22 (1.71)	0.006
Mean HAS-BLED score (SD)	2.30 (1.14)	2.29 (1.12)	0.009	2.25 (1.15)	2.25 (1.16)	0.000	2.43 (1.15)	2.43 (1.12)	0.000

Values are expressed as numbers (percent), unless otherwise stated.

NSAIDs, non-steroidal anti-inflammatory drugs; SD, standard deviation; SE, systemic embolism; SMD, absolute standardized mean difference.

### Dabigatran–rivaroxaban-matched cohort

The median follow-up time was 18.6 months for dabigatran and 18.2 months for rivaroxaban. In the dabigatran group, stroke/SE occurred with an event rate of 1.84/100 person-years compared with 2.21/100 person-years in the rivaroxaban group [HR 0.88; 95% confidence interval (CI) 0.76–1.02]. A major bleeding event occurred at a rate of 1.40/100 person-years in the dabigatran group, and 1.93 in the rivaroxaban group (HR 0.75; 95% CI 0.64–0.88).

### Dabigatran–apixaban-matched cohort

The median follow-up time was 18.2 months for dabigatran users and 12.2 months for apixaban users. Among dabigatran users, stroke/SE occurred at a rate of 1.83/100 person-years, while the event rate was 2.62/100 person-years for apixaban users (HR 0.88; 95% CI 0.75–1.02). Major bleeding occurred at an event rate of 1.38/100 person-years in the dabigatran group vs. 1.54/100 person-years in the apixaban group (HR 1.03 95% CI 0.85–1.24). The risk of GI bleeding was significantly higher for dabigatran with event rates of 3.22/100 person-years vs. 2.17/100 person-years in the apixaban group (HR 1.48; 95% CI 1.28–1.70).

### Apixaban–rivaroxaban-matched cohort

The median follow-up time was 18.1 months in the rivaroxaban group, and 12.5 months in the apixaban group. The event rate of stroke/SE was 2.65/100 person-years for the apixaban group vs. 2.31/100 person-years for the rivaroxaban group (HR 1.00; 95% CI 0.89–1.14). The event rates of major bleeding were 1.76/100 person-years vs. 2.10/100 person-years in the apixaban- and rivaroxaban groups, respectively (HR 0.79; 95% CI 0.68–0.91).

### Subgroup analyses

**Figure 4:**
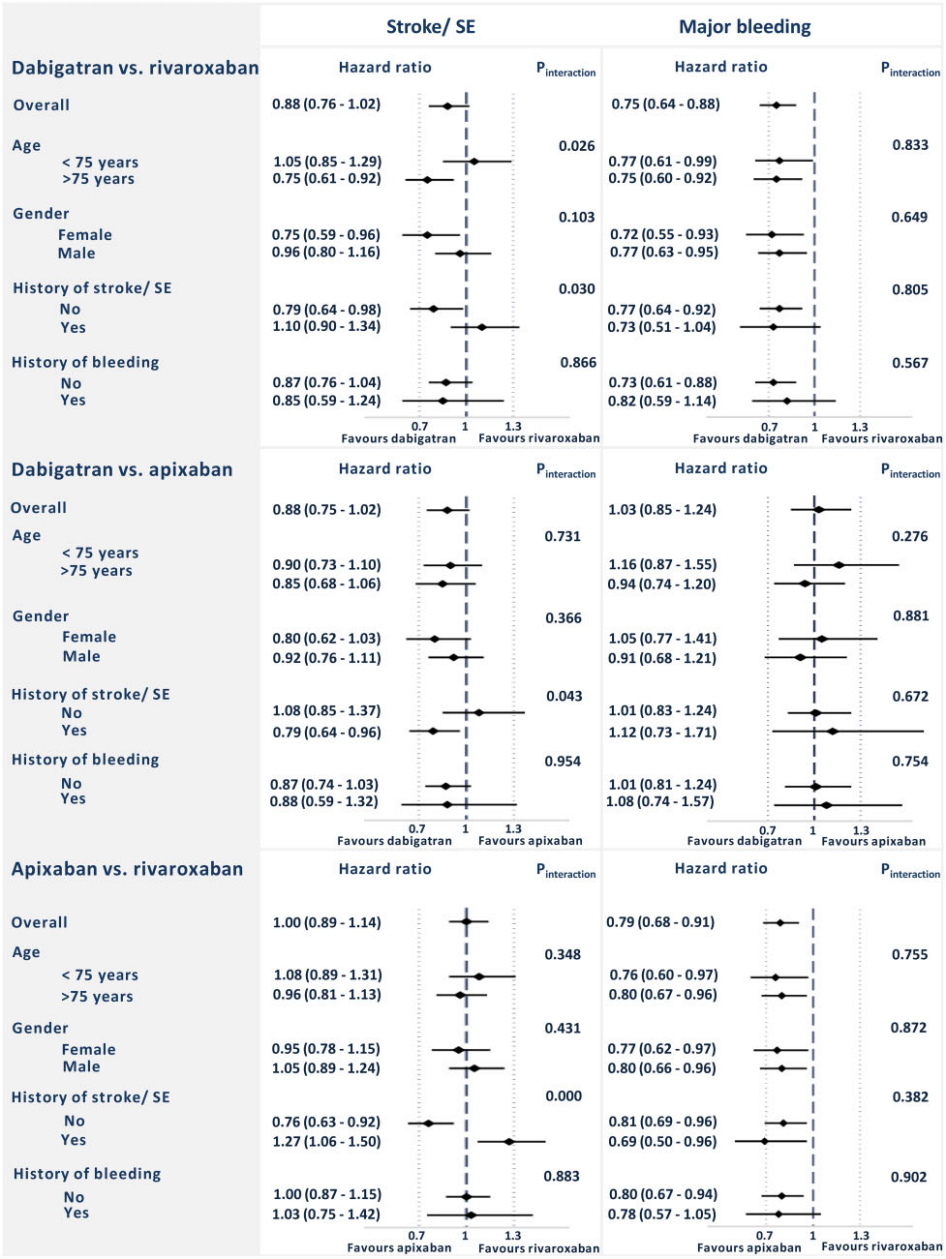
The risk of stroke or systemic embolism and major bleeding in selected subgroups. SE, systemic embolism.

The risks of stroke or SE and major bleeding in selected subgroups are shown in *Figure 4*. No significant heterogeneity between subgroups was found with respect to risk of major bleeding. In the dabigatran–rivaroxaban-matched cohort, significant heterogeneity regarding risk of stroke/SE was seen in two subgroups; namely age <75 vs. >75 years, and patients with or without prior stroke/SE. Also, in the two other cohorts, heterogeneity was seen with respect to risk of stroke/SE in the subgroup of patients with or without prior stroke/SE.

**Figure 5:**
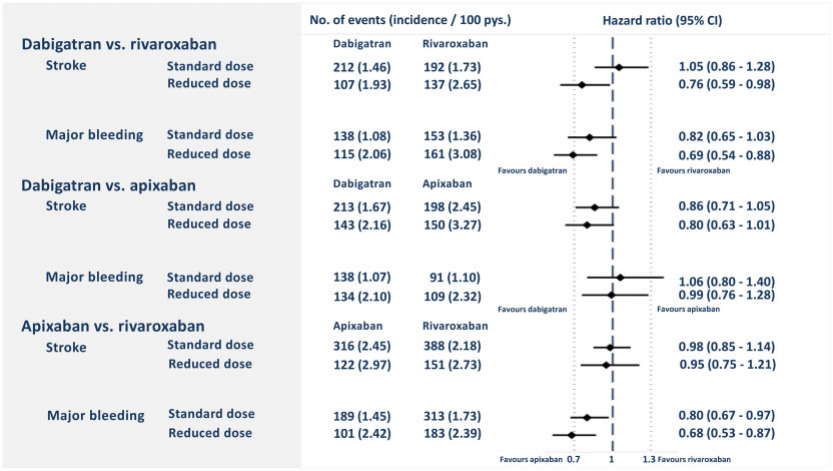
The risk of stroke or systemic embolism and major bleeding for patients using standard or reduced dose non-vitamin K antagonist oral anticoagulants. CI, confidence interval; Pys., person-years.

Patients initiating standard or reduced dose NOACs differed in baseline characteristics; the patients receiving reduced doses were more likely to be older and having more comorbidities than patients starting standard doses (Supplementary material online, *Table S5*). After propensity score re-matching on initial doses, both reduced- and standard-dose patients showed broadly consistent results to the main analysis (*Figure 5*).

### Sensitivity analyses

The results of the sensitivity analyses are shown in Supplementary material online, *Table S6* and were in line with the primary analyses.

### Non-vitamin K antagonist oral anticoagulant–warfarin comparisons

Comparing each NOAC with warfarin, we found no significant differences in the adjusted HRs of stroke/SE for any NOAC compared with warfarin, while dabigatran and apixaban were both associated with lower risk of major bleeding (Supplementary material online, *Table S4*).

## Discussion

In this study, we compared the risks of stroke or SE and major bleeding associated with use of dabigatran, rivaroxaban, and apixaban in a large nationwide cohort of anticoagulant-naïve patients with AF. In propensity score-matched analyses, we found no statistically significant differences in the risk of stroke or SE between NOACs, but dabigatran and apixaban were associated with significantly lower risk of major bleeding compared with rivaroxaban. The reduction of bleeding risk associated with dabigatran and apixaban was consistent for CRNM bleeding, major or CRNM bleeding, and intracranial bleeding. Dabigatran and rivaroxaban were associated with a significantly higher risk of GI bleeding compared with apixaban.

Clinical trials and recent meta-analyses have shown that the NOACs are at least as effective as warfarin in stroke prevention and are associated with a similar or reduced risk of bleeding.^4–6^^,^^18^^,^^19^ In registry-based observational studies comparing the NOACs with warfarin, very similar results have been found.^20–23^ As the proportion of patients with AF being started on a NOAC instead of warfarin is increasing,^24^ knowledge of the comparative effectiveness and safety profiles of the different NOACs in clinical practice is needed.

Our study is one of very few studies designed to directly compare the effectiveness and safety of three individual NOACs in clinical practice. As treatment with NOACs is the standard of care in AF today,^2^ such a comparison seems more relevant for the practicing clinician.

The NOACs were examined pairwise in PSM analyses. A strength of our study is the inclusion of all anticoagulant-naïve new users of a NOAC from a nationwide cohort; this should eliminate selection and participation bias often present in observational cohort studies. Furthermore, the follow-up times were longer and the number of patients included in the matched cohorts larger in our study compared with most previous studies.^23^^,^^25^

Our current findings are in line with similar studies.^23^^,^^25–27^ In a recent Danish study by Staerk *et al*.,^27^ including 31 522 patients with AF, multivariate Cox regression was chosen over PSM. In line with our findings, dabigatran and apixaban were associated with lower bleeding risk compared with rivaroxaban, but no significant differences were seen between the NOACs in terms of effectiveness. In another Danish study by Andersson *et al*.,^25^ including 12 638 new users of NOACs, PSM was performed, and no significant differences in associated risk of stroke/SE or major bleeding were found between NOACs. However, due to the low number of patients in each matched cohort, this study might have been underpowered. Similarly, Noseworthy *et* *al*.^26^ found no significant differences in effectiveness between the NOACs in their PSM cohorts, and both dabigatran and apixaban were associated with significantly lower bleeding risk compared with rivaroxaban. In the largest observational study to date, Lip *et* *al*.^23^ studied 285 292 patients pooled from the US Centers for Medicare and Medicaid Services Medicare data and four commercial claims databases in the USA (the ARISTOPHANES study). After PSM of patients with AF treated with a NOAC, apixaban was associated with significantly lower risk of both stroke or SE and major bleeding compared with dabigatran and rivaroxaban. Dabigatran compared with rivaroxaban was associated with a similar risk of stroke/SE but significantly lower risk of bleeding. A major limitation of the ARISTOPHANES study was the very short median follow-up time in all cohorts of just over 4 months. Another limitation involves the use of healthcare claims databases, necessitating Medicare or Medicaid eligibility for patient inclusion and relying on billing codes to define all baseline characteristics and outcomes. This increases risk of selection bias and loss to follow-up bias.

Our *post* *hoc* analysis comparing NOACs with warfarin were also generally in line with the results from similar real-world studies,^20–23^ showing non-significant differences in the risk of stroke/SE associated with NOACs, and significantly lower risks of major bleeding for both dabigatran and apixaban. Comparing our results with the RCTs,^4–6^ we did not find the reductions in stroke risk with dabigatran 150 mg and apixaban compared with warfarin that was shown in the RE-LY and ARISTOTLE trials.^5^^,^^6^ This has, however, been the case in several previous real-world studies.^21–23^ Minor discrepancies from the RCTs are to be expected, since these are not randomized comparisons. Despite adjustments, remaining unmeasured confounders will always exist.

In the subgroup analyses performed in our study, significant interactions were seen between groups using NOACs as primary or secondary stroke prophylaxis. These findings are difficult to explain. Since they represent interactions based on subgroup analyses of non-randomized comparisons, they are most likely due to chance. The risks of stroke/SE and major bleeding in the cohorts rematched on standard and reduced doses were broadly consistent with the main findings.

### Strengths and limitations

There are fundamental differences between observational studies and RCTs, where the higher event rates often seen in registry studies reflect some of these differences.^4–6^^,^^28^^,^^29^ Inclusion of data into the nationwide registries is mandatory in Norway; this eliminates selection, participation, and recall bias. It also ensures a study population large enough for robust calculations. These advantages of nationwide registries are summarized in a recent position document from the European Heart Rhythm Association.^30^

The Norwegian system of general reimbursement of medical expenses for the treatment of serious and prolonged chronic illnesses ensures that all patients included in the study are in fact using OACs for AF, and not venous thromboembolism or any other condition; a challenge for similar studies based on registries where information on indication for treatment is unavailable.^31^^,^^32^

A well-known limitation is that conventional multivariate regression, as well as PSM cannot control for unknown or unmeasurable confounders.^33^ In the total study population, before PSM was performed, patients starting rivaroxaban and apixaban were generally older and sicker than patients starting dabigatran (Supplementary material online, *Table S3*). It seems likely that the patients starting rivaroxaban and apixaban could also have other comorbidities or underlying factors that we have not taken into account, as well as a higher degree of frailty; an element which is difficult to measure in this type of study based on nationwide administrative registries, but which in this case likely is driving the estimates in favour of dabigatran.

The events recorded were not adjudicated. There was also very likely a certain degree of miscoding and under-reporting of comorbidities and events. Despite nationwide inclusion of patients, because of demographics the study participants were still largely White northern Europeans. This may limit the generalizability of the results. Another limitation is that the registries do not supply information on relevant laboratory analyses such as estimated glomerular filtration rate, cardiac troponins, erythrocyte count, thrombocyte count, or liver enzymes; or other important patient characteristics such as body weight, lifestyle, or smoking habits.

Dabigatran was the first, rivaroxaban the second, and apixaban the third NOAC available in Norway, and all drugs were available in the whole study period. The proportion of patients starting on apixaban increased steadily throughout the study period (Supplementary material online, *Table S3*). Temporal changes in prescription patterns for NOACs might influence the number of events in each group. However, we found no significant differences between the NOACs regarding associated risk of stroke/SE; and dabigatran (the first NOACs on the market) and apixaban (the last NOAC on the market) were both associated with significantly lower risks of major bleeding compared with rivaroxaban (the second NOAC on the market). In addition, we created well-balanced cohorts in terms of risk factors; thus, it seems unlikely that temporal changes have played any important role for our results. To account for the approximately 6 months average shorter follow-up time for apixaban compared with dabigatran and rivaroxaban we performed a separate sensitivity analysis restricting the follow-up time to 12 months with results in line with the main analyses.

Evaluation of the appropriateness of the dose prescribed (standard or reduced dose of NOAC) requires knowledge not only of patient age but also of serum creatinine and body weight. The variables serum creatinine and body weight are unfortunately not available from the nationwide registries in Norway, like in many other registries.^23^^,^^25–27^ Although we were unable to identify users of NOACs per label regarding dose, we have attempted to compensate for this by performing *de novo* propensity score estimation and matching within dosage groups. Furthermore, based on a recent study from UK, there are reasons to believe that the majority of AF patients are prescribed appropriate doses of NOACs; the UK study found between 75% and 85% of patients to be appropriately dosed.^34^

We studied drug exposure at the level of pharmacy dispensation and have no information on patient’s real intake of OAC. However, it is unlikely to expect any differences between groups in this respect.

Due to the limitations of our study, the results should be interpreted with caution and need to be confirmed by findings from NOAC vs. NOAC RCTs. This is especially the case for the subgroup analyses, where we after careful consideration did not adjust for multiple comparisons (e.g. Bonferroni correction).

## Conclusion

In this large registry-based study including 65 563 anticoagulant-naïve patients with AF initiating OAC therapy, we found no statistically significant differences in risk of stroke or SE between dabigatran, rivaroxaban, and apixaban, while both dabigatran and apixaban were associated with significantly lower risks of major bleeding compared with rivaroxaban.

## Funding

This work was supported by the South-Eastern Norway Regional Health Authority. The study received additional support through the Bristol-Myers Squibb/Pfizer-sponsored European Investigator Initiated research Program (ERISTA) [Grant number 2016-ELI-0407].

**Conflict of interest:** O.-C.R. reports personal fees from Merck, Bayer, Boehringer Ingelheim, Novartis, and Novo Nordisk, outside the submitted work. C.J. reports personal fees from BMS/Pfizer and Bayer, outside the submitted work. W.G. reports grants and personal fees from Bayer, MSD, Novartis, and Amgen, outside the submitted work. S.H. reports personal fees from Bristol-Myers Squibb, Bayer, Boehringer Ingelheim, Pfizer, Merck, and Daiichi-Sankyo, outside the submitted work.

## Supplementary Material

pvz086_Supplementary_Data
