# Supplementary material for: Comparison of dabigatran, rivaroxaban, and apixaban for effectiveness and safety in atrial fibrillation: a nationwide cohort study
Source: Eur Heart J Cardiovasc Pharmacother. 2020 Jan 14;6(2):75–85. doi: 10.1093/ehjcvp/pvz086 (PMC7073510; doi:10.1093/ehjcvp/pvz086)
Supplement: pvz086_Supplementary_Data [file pvz086_supplementary_data.docx]

**Supplementary table S1**

**ICD-10 (International Classification of Diseases, 10th revision) and NOMESCO (Nordic Medico-Statistical Committee) codes used in definitions of co-morbidities and outcomes. Comorbidities were recognized either by ICD-10 diagnoses from hospital stays, or by a combination of hospital diagnoses and drugs dispensed. ATC (Anatomical Therapeutic Chemical system ) codes from NorPD identified disease-specific drugs (e.g. anti-diabetics) and ICD-10 or International Classification for Primary Care 2 (ICPC-2) codes used as reasons for reimbursement of drugs for chronic illnesses for less specific drugs (e.g. beta blockers).**

| **Conditions** | **ICD-10 code or procedure codes (NOMESCO) from NPR** | **ATC code or reimbursement code in NorPD** |
| --- | --- | --- |
| Atrial fibrillation | I48 | Reimbursement code: I48 , K78 (ICPC) |
| Additional diagnoses to identify “valvular atrial fibrillation” | ICD10: I050, I052, I342, Z952  NOMESCO codes: FKD00, FKA, FMD00, |  |
| Hypertension | I10, I11, I12, I13, I15 | Reimbursement codes: I10-I13, I15 (ICD10) or K86, K87 (ICPC) |
| Chronic kidney disease | N181, N182, N183, N184, N185, N189, N19 |  |
| Ischemic heart disease | I20, I21, I22, I23, I24, I25 |  |
| Heart failure | I500, I501, I509 | Reimbursement codes: I50 (ICD10) or K77 (ICPC) |
| Diabetes | E10, E11, E12, E13 | ATC code A10A or A10B |
| Chronic lower respiratory tract disorders | J40 – J47 | Reimbursement codes: J44 , J45 (ICD10) or R95 (ICPC |
| Active cancer | C00, C01, C02, C03, C04, C05, C06, C07, C08, C09, C10, C11, C12, C13, C14, C15, C16, C17, C18, C19, C20, C21, C22, C23, C24, C25, C26, C30, C31, C32, C33, C34, C37, C38, C39,  C40, C41, C43, C44, C45, C46, C47, C48, C49, C50, C51, C52, C53, C54, C55, C56, C57, C58, C60, C61, C62, C63, C64, C65, C66, C67, C68, C69, C70, C71, C72, C73, C74, C75, C76, C77, C78, C79, C80, C81, C82, C83, C84, C85, C86, C88, C90, C91, C92, C93, C94, C95, C96, C97 |  |
| Peripheral artery disease | I70, I71, I72, I73, I74,  I77, I78, I79 |  |
| Inflammatory polyarthropathies | M05 – M14 |  |
| Ischaemic stroke | I630, I631, I632, I633, I634, I635, I636, I638, I639, I64 |  |
| Transient ischaemic attack (TIA) | G450, G451, G452, G453, G454, G458, G459, G46 |  |
| Ischaemic or haemorrhagic stroke | I600, I601, I602, I603, I604, I605, I606, I607, I608, I609, I610, I611, I612, I613, I614, I615, I616, I618, I619, I620, I621, I629, I630, I631, I632, I633, I634, I635, I636, I638, I639, I64, |  |
| Major bleeding | K920, K921, I600-I609, I610-I619,  I620-I629, I230, I312, M250, H431,  H356, H313, H450, J942, K661  *Addition: A CRNM-bleeding diagnosis will be converted to a major bleeding diagnose if blood transfusion (NCMP REGG00, RXGG02) is coded within 10 days.* |  |
| Systemic embolism | I74 |  |
| Intracranial bleeding | I600, I601, I602, I603, I604, I605, I606, I607, I608, I609, I610, I611, I612, I613, I614, I615, I616, I618, I619, I620, I621, I629 |  |
| Gastrointestinal bleeding | K920, K921, K922, K250, K252, K254, K256, K260, K262, K264, K266, K270, K272, K274, K276, K280, K282, K284, K286, K625, K228, K221, K290, K528, K625, I850 |  |
| CRNM bleeding | K922, K250, K252, K254, K256, K260, K262, K264, K266, K270, K272, K274, K276, K280, K282, K284, K286, K625, K228, K221, K290, K528, K625, I850, H113, R040, R041, R042, R048, R049, N836, N837, N920, N921, N922, N923, N924, N925, N926, N930, N938, N939, A985, N421, N857, N921, O721, S064, S065, S066, S068, T140, T141, T142, T143, T144, T145, T146, T147, T148, T149, D683, D698, D699, N02, R31, R58, D62 |  |
| Anaemia | D50, D51, D52, D53, D55, D56, D57, D58, D59, D60, D61, D62, D63,D64 |  |
| Alcoholism | E244, E52, G312, G621, G721, I426, K70, K860, O354, T51, Z714, Z721 |  |
| Use of NSAID |  | M01A |
| Use of antiplatelet drugs |  | B01A C |
| Use of cholesterol lowering drugs |  | C10A, C10B |

NPR, Norwegian Patient Registry; NorPD,Norwegian Prescription Database; NCMP, Norwegian Classification of Medical Procedures

**Supplementary table S2; ICD-codes used to calculate risk scores**

| **CHADS2-VASC** | | |
| --- | --- | --- |
| **Point** | **Condition** | **Definition** |
| 1 | Heart Failure | use definition from baseline covariates (Table 1) |
| 1 | Hypertension | use definition from baseline covariates (Table 1) |
| 1 | Diabetes mellitus | use definition from baseline covariates (Table 1) |
| 2 | Stroke, TIA or systemic embolism | use definition from baseline covariates (Table 1) |
| 1 | Vascular Disease (myocardial infarction or peripheral arterial disease) | Combined definitions from baseline covariates “Ischaemic Heart Disease” , and “Vascular disease” in table 1. |
| 1 | Female | |
| 1 | Age 65-<75 years | |
| 2 | Age≥ 75 years | |
| **HAS-BLED** | | |
| **Point** | Condition | **Definition** |
| **1** | Hypertension | Use definition for "Hypertension” from baseline comorbidities |
| **1** | Abnormal kidney function | Use definition for "Chronic kidney disease" from baseline comorbidities |
| **1** | Abnormal liver function: | Use definition for "Liver disease" from baseline comorbidities |
| **1** | Stroke, TIAor TIA | use definition “History of stroke” from baseline comorbidities |
| **1** | Any bleeding other than haemorrhagic stroke | Use definition of Major and CRNM bleeding from baseline comorbidities, excluding codes for haemorrhagic stroke I60, I61, I690-I692 |
| N/A | Labile INR | Not available |
| **1** | Age≥ 65 years | 1 point for age 65 years or older |
| **1** | Alcohol/ Drug Therapy | Use definition of "Alcoholism" , “Use of NSAIDs last 12 months” and “Use of antiplatelet drugs last 12 months, from baseline comorbidities. |

Values are numbers (percent) unless otherwise specified. TIA, transient ischaemic attack; NSAIDs, non-steroidal inti-inflammatory drugs; INR, International Normalised Ratio

**Supplementary table S3. Baseline characteristics of total study population**

|  | Dabigatran  n= 10 413 | Apixaban  n= 28 363 | Rivaroxaban n= 13 700 | Warfarin n=13 087 | Total  n= 65 563 |
| --- | --- | --- | --- | --- | --- |
| Year of entry into study  2013  2014  2015  2016  2017 | 4 476 (43.0)  3 219 (30.9)  1 106 (10.6)  837 (8.0)  775 (7.4) | 371 (1.3)  3 438 (12.1)  6 751 (23.8)  8 562 (30.2)  9 241 (32.6) | 2 995 (21.9)  2 980 (21.8)  3 230 (23.6)  2 736 (20.0)  1 759 (12.8) | 6 078 (46.4)  3 648 (27.9)  1 898 (14.5)  966 (7.4)  497 (3.8) | 13 920 (21.2)  13 285 (20.3)  12 985 (19.8)  13 101 (20.0)  12 272 (18.7) |
| OAC dose  Standard  Reduced | 6 652 (63.9)  3 761 (36.1) | 21 149 (74.6)  7 214 (25.4) | 10 363 (75.6)  3 337 (24.4) | 13 087 (100) | 51 251 (78.2)  14 312 (21.8) |
| Age |  |  |  |  |  |
| Mean (SD) | 70.6 (11.2) | 73.76 (11.3) | 72.7 (11.1) | 73.4 (12.1) | 73.0 (11.4) |
| Median (25th –  75^th^ percentile) | 71 (64 – 79) | 74 (67 – 82) | 73 (66 – 81) | 75 (66 – 83) | 73 (66 – 81) |
| <65 yrs. | 2 687 (25.8) | 5 267 (18.6) | 2 787 (20.3) | 2 744 (21.0) | 13 508 (20.6) |
| 65 to 74 yrs. | 3 869 (37.2) | 9 310 (32.8) | 4 805 (35.1) | 3 693 (28.2) | 21 723 (33.1) |
| ≥ 75 yrs. | 3 857 (37.0) | 13 786 (48.6) | 6 108 (44.6) | 6 650 (50.8) | 30 439 (46.4) |
| Male sex | 6 433 (61.8) | 15 890 (56.0) | 7 944 (58.0) | 7 923 (60.5) | 38 258 (58.3) |
| Hypertension | 6 693 (64.3) | 19 234 (67.8) | 9 289 (67.8) | 9 222 (70.5) | 44 506 (67.8) |
| Ischaemic heart disease | 2 119 (20.3) | 6 979 (24.6) | 3 061 (22.3) | 4 557 (34.8) | 16 733 (25.5) |
| Vascular disease | 1 308 (12.6) | 4 884 (17.2) | 2 065 (15.1) | 3 207 (24.5) | 11 487 (17.5) |
| Heart failure | 2 140 (20.6) | 7 147 (25.2) | 3 043 (22.2) | 4 593 (35.1) | 16 940 (25.8) |
| History of stroke | 1 356 (13.0) | 3 822 (13.5) | 1 792 (13.1) | 1 720 (13.1) | 8 696 (13.2) |
| Chronic Kidney Disease | 245 (2.4) | 1 991 (7.0) | 627 (4.6) | 1 634 (12.5) | 4 502 (6.9) |
| Diabetes Mellitus | 1 324 (12.7) | 4 189 (14.8) | 1 887 (13.8) | 2 282 (17.4) | 9 692 (14.8) |
| Inflammatory polyarthropathies | 474 (4.6) | 1 532 (5.4) | 678 (4.9) | 897 (6.9) | 3 584 (5.5) |
| COPD | 2 500 (24.0) | 7 660 (27.0) | 3 529 (25.8) | 3 383 (25.9) | 17 102 (26.0) |
| Active cancer (diagnosis last 12 months) | 770 (7.4) | 2 774 (9.8) | 1 263 (9.2) | 1 307 (10.0) | 6 119 (9.3) |
| History of anaemia | 458 (4.4) | 2 126 (7.5) | 757 (5.5) | 1 143 (8.7) | 4 489 (6.8) |
| History of bleeding | 1 144 (11.0) | 3 915 (13.8) | 1 715 (12.5) | 2 022 (15.5) | 8 804 (13.4) |
| Use of antiplatelet drugs last 12 months | 5 125 (49.2) | 14 380 (50.7) | 7 208 (52.6) | 6 930 (53.0) | 33 700 (51.3) |
| Use of NSAIDS last 12 months | 2 512 (24.1) | 6 198 (21.9) | 3 148 (23.0) | 2 657 (20.3) | 14 538 (22.1) |
| Use of cholesterol lowering drugs | 4 629 (44.5) | 13 863 (48.9) | 6 315 (46.1) | 6 834 (52.2) | 31 702 (48.3) |
| Mean CHA2DS2 VaSc – score (SD) | 2.9 (1.7) | 3.3 (1.7) | 3.1 (1.7) | 3.4 (1.8) | 3.2 (1.7) |
| Men HAS-BLED – score (SD) | 2.2 (1.1) | 2.3 (1.1) | 2.4 (1.1) | 2.5 (1.1) | 2.3 (1.1) |

Values are numbers (percent) unless otherwise specified. SD, standard deviation; TIA, transient ischaemic attack; NSAIDs, non-steroidal inti-inflammatory drugs.

**Supplementary table S4. NOACs compared with warfarin. Number of events, crude incidence rates and hazard ratios of stroke/SE and major bleeding.**

|  |  | **No of events (incidence / 100 person years)** | **Hazard ratio (95% CI)*** |
| --- | --- | --- | --- |
| **Stroke/SE** |  |  |  |
| Warfarin |  | 519 (2.20) | Ref. |
| Dabigatran | Standard and reduced dose | 360 (1.83) | 0.94 (0.82 - 1.08) |
|  | 150 mg bid. | 213 (1.67) | 0.97 (0.82 – 1.15) |
|  | 110 mg bid. | 147 (2.13) | 0.89 (0.73 – 1.07) |
| Rivaroxaban | Standard and reduced dose | 541 (2.31) | 1.03 (0.91 - 1.16) |
|  | 20 mg od. | 388 (2.18) | 1.05 (0.91 – 1.20) |
|  | 15 mg od. | 153 (2.75) | 1.08 (0.90 - 1.30) |
| Apixaban | Standard and reduced dose | 941 (2.70) | 1.04 (0.94 - 1.17) |
|  | 5 mg bid. | 641 (2.44) | 1.02 (0.90 – 1.15) |
|  | 2.5 mg bid. | 300 (3.50) | 1.10 (0.95 - 1.28) |
| **Major bleeding** |  |  |  |
| Warfarin |  | 607 (2.51) | Ref. |
| Dabigatran | Standard and reduced dose | 275 (1.38) | 0.74 (0.64 - 0.86) |
|  | 150 mg bid. | 138 (1.07) | 0.69 (0.56 – 0.84) |
|  | 110 mg bid. | 137 (1.97) | 0.82 (0.68 – 0.99) |
| Rivaroxaban | Standard and reduced dose | 496 (2.10) | 0.97 (0.86 - 1.10) |
|  | 20 mg od. | 313 (1.73) | 0.89 (0.77 – 1.03) |
|  | 15 mg od. | 183 (3.27) | 1.17 (0.99 - 1.39) |
| Apixaban | Standard and reduced dose | 673 (1.90) | 0.76 (0.68 - 0.85) |
|  | 5 mg bid. | 418 (1.57) | 0.73 (0.64 – 0.83) |
|  | 2.5 mg bid. | 255 (2.92) | 0.85 (0.73 - 0.99) |

Abbreviations: NOAC, non-vitamin K antagonist oral anticoagulant; SE, systemic embolism; CI, confidence interval; od, omne die (once daily); bid, bis in die (twice daily).

*Multivariate Cox proportional hazards regression using the same 16 covariates as in the propensity score matched main analyses: age, gender, chronic kidney disease, hypertension, diabetes, ischaemic heart disease, peripheral artery disease (PAD), heart failure, history of stroke/SE, history of bleeding-related hospitalisation, anaemia, active cancer (cancer diagnosis last 12 months), chronic lower respiratory tract disease, use of cholesterol lowering drugs, use of antiplatelet drugs and use of NSAIDs during the last 12 months.

Standard and reduced doses of NOACs are analysed together.

**Supplementary tables S5. Baseline characteristics after propensity score matching of patients using standard and reduced dose NOACs separately**

1. **Baseline characteristics, propensity matched groups, standard dose NOACs**

|  | **Dabigatran – rivaroxaban matched cohort**  **n= 13 076** | | | **Dabigatran – apixaban matched cohort**  **n= 13 304** | | | **Apixaban - rivaroxaban matched cohort**  **n= 20 726** | | |
| --- | --- | --- | --- | --- | --- | --- | --- | --- | --- |
|  | **Dabigatran,**  **n= 6 538** | **Rivaroxaban**  **n= 6 538** | **SMD** | **Dabigatran**  **n= 6 652** | **Apixaban**  **n= 6 652** | **SMD** | **Apixaban**  **n=10 363** | **Rivaroxaban**  **n=10 363** | **SMD** |
| **Age**  **Mean (SD)**  **Median**  **< 65 yrs.**  **65-74 yrs.**  **≥ 75 yrs.** | 65.9 (9.0)  67  2 375 (36.3)  3 232 (49.4)  931 (14.2) | 65.9 (9.5)  67  2 466 (37.7)  3 020 (46.2)  1 052 (16.1) | 0.004 | 65.6 (9.3)  67  2 489 (37.4)  3 232 (48.6)  931 (14.0) | 65.7 (10.0)  67  2 624 (39.4)  2 899 (43.6)  1 129 (17.0) | 0.013 | 70.4 (10.4)  71  2 527 (24.4)  4 190 (40.4)  3 646 (35.2) | 70.4 (10.5)  71  2 574 (24.8)  4 159 (40.1)  3 630 (35.0) | 0.006 |
| **Male gender** | 4 533 (69.3) | 4 537 (69.4) | 0.001 | 4 644 (69.8) | 4 664 (70.1) | 0.007 | 6 266 (60.5) | 6 304 (60.8) | 0.008 |
| **Hypertension** | 3 844 (58.8) | 3 910 (59.8) | 0.022 | 3 869 (58.2) | 3 805 (57.2) | 0.020 | 6 663 (64.3) | 6 669 (64.4) | 0.003 |
| **Ischaemic heart disease** | 1 045 (16.0) | 1 024 (15.7) | 0.002 | 1 058 (15.9) | 1 051 (15.8) | 0.002 | 1 968 (19.0) | 1 937 (18.7) | 0.003 |
| **Vascular disease** | 366 (5.6) | 324 (5.0) | 0.015 | 368 (5.5) | 359 (5.4) | <0.001 | 852 (8.2) | 805 (7.8) | 0.009 |
| **Heart failure** | 982 (15.0) | 961 (14.7) | 0.011 | 1 002 (15.1) | 949 (14.3) | 0.023 | 1 830 (17.7) | 1 819 (17.6) | 0.005 |
| **Chronic kidney disease** | 60 (0.9) | 46 (0.7) | 0.022 | 60 (0.9) | 50 (0.8) | 0.015 | 225 (2.2) | 207 (2.0) | 0.011 |
| **Diabetes mellitus** | 784 (12.0) | 804 (12.3) | 0.009 | 789 (11.9) | 797 (12.0) | 0.003 | 1 418 (13.7) | 1 369 (13.2) | 0.012 |
| **Chronic lower respiratory tract diseases** | 1 538 (23.5) | 1 634 (25.0) | 0.001 | 1 549 (23.3) | 1 624 (24.4) | 0.006 | 2 696 (26.0) | 2 689 (25.9) | 0.002 |
| **Active cancer (diagnosis last 12 months)** | 382 (5.8) | 377 (5.8) | 0.001 | 383 (5.8) | 398 (6.0) | 0.009 | 853 (8.2) | 866 (8.4) | 0.007 |
| **History of stroke /TIA** | 691 (10.6) | 672 (10.3) | 0.011 | 698 (10.5) | 726 (10.9) | 0.012 | 1 296 (12.5) | 1 272 (12.3) | 0.006 |
| **History of anaemia** | 173 (2.6) | 157 (2.4) | 0.016 | 174 (2.6) | 128 (1.9) | 0.046 | 443 (4.3) | 423 (4.1) | 0.009 |
| **History of bleeding** | 579 (8.9) | 583 (8.9) | 0.003 | 580 (8.7) | 540 (8.1) | 0.022 | 1 222 (11.8) | 1 151 (11.1) | 0.021 |
| **Use of antiplatelet drugs last 12 months** | 2 863 (43.8) | 2 884 (44.1) | 0.006 | 2 875 (43.2) | 2 855 (42.9) | 0.006 | 5 182 (50.0) | 5 145 (49.6) | 0.007 |
| **Use of NSAIDS last 12 months** | 1 697 (26.0) | 1 703 (26.0) | 0.002 | 1 714 (25.8) | 1 772 (26.6) | 0.020 | 2 538 (24.5) | 2 470 (23.8) | 0.015 |
| **Use of cholesterol lowering drugs** | 2 721 (41.6) | 2 747 (42.0) | 0.008 | 2 740 (41.2) | 2 761 (41.5) | 0.006 | 4 562 (44.0) | 4 587 (44.3) | 0.005 |
| **Mean CHA2DS2 VaSc – score (SD)** | 2.3 (1.5) | 2.3 (1.4) | 0.004 | 2.3 (1.5) | 2.3 (1.5) | 0.001 | 3.0 (1.7) | 2.9 (1.6) | 0.015 |
| **Mean HAS-BLED – score (SD)** | 2.0 (1.1) | 2.0 (1.1) | 0.005 | 2.0 (1.1) | 2.0 (1.1) | 0.023 | 2.3 (1.2) | 2.3 (1.2) | 0.011 |

Values are numbers (percent) unless otherwise specified. SD, standard deviation; SMD, absolute standardised mean difference; TIA, transient ischaemic attack; NSAIDs, non-steroidal inti-inflammatory drugs.

1. **Baseline characteristics, propensity matched groups, reduced dose NOACs**

|  | **Dabigatran – rivaroxaban matched cohort**  **n= 6 148** | | | **Dabigatran – apixaban matched cohort**  **n= 7 276** | | | **Apixaban - rivaroxaban matched cohort**  **n= 6 640** | | |
| --- | --- | --- | --- | --- | --- | --- | --- | --- | --- |
|  | **Dabigatran,**  **n= 3 074** | **Rivaroxaban**  **n= 3 074** | **SMD** | **Dabigatran**  **n= 3 638** | **Apixaban**  **n= 3 638** | **SMD** | **Apixaban**  **n=3 320** | **Rivaroxaban**  **n= 3 320** | **SMD** |
| **Age**  **Mean (SD)**  **Median**  **< 65 yrs.**  **65-74 yrs.**  **≥ 75 yrs.** | 79.8 (8.3)  81  150 (4.9)  510 (16.6)  2 414 (78.5) | 79.8 (9.6)  81  204 (6.6)  594 (19.3)  2 276 (74.0) | 0.003 | 80.0 (8.3)  81  180 (4.9)  566 (15.6)  2 892 (79.5) | 80.0 (9.2)  82  233 (6.4)  638 (17.5)  2 767 (76.1) | 0.023 | 80.0 (9.8)  82  248 (7.5)  591 (17.8)  2 481 (74.7) | 80.0 (9.5)  82  203 (6.1)  640 (19.3)  2 477 (74.6) | 0.002 |
| **Male gender** | 1 466 (47.7) | 1 469 (47.8) | 0.002 | 1 700 (46.7) | 1 730 (47.6) | 0.017 | 1 656 (49.9) | 1 624 (48.9) | 0.019 |
| **Hypertension** | 2 329 (75.8) | 2 350 (76.4) | 0.017 | 2 733 (75.1) | 2 738 (75.3) | 0.001 | 2 563 (77.2) | 2 576 (77.6) | 0.012 |
| **Ischaemic heart disease** | 868 (28.2) | 862 (28.0) | 0.001 | 974 (26.8) | 967 (26.6) | 0.001 | 1 020 (30.7) | 992 (29.9) | 0.019 |
| **Vascular disease** | 334 (10.9) | 324 (10.5) | 0.001 | 352 (9.7) | 356 (9.8) | 0.011 | 385 (11.6) | 376 (11.3) | 0.006 |
| **Heart failure** | 1 034 (33.6) | 1 032 (33.6) | 0.003 | 1 088 (29.9) | 1 044 (28.7) | 0.027 | 1 228 (37.0) | 1 198 (36.1) | 0.021 |
| **Chronic kidney disease** | 185 (6.0) | 182 (5.9) | 0.003 | 185 (5.1) | 182 (5.0) | 0.003 | 413 (12.5) | 414 (12.5) | <0.001 |
| **Diabetes mellitus** | 427 (13.9) | 442 (14.4) | 0.012 | 511 (14.0) | 501 (13.8) | 0.010 | 519 (15.6) | 507 (15.3) | 0.009 |
| **Chronic lower respiratory tract diseases** | 785 (25.5) | 761 (24.8) | 0.005 | 926 (25.5) | 953 (26.2) | 0.006 | 883 (26.6) | 831 (25.0) | 0.002 |
| **Active cancer (diagnosis last 12 months)** | 300 (9.8) | 325 (10.6) | 0.020 | 359 (9.9) | 371 (10.2) | 0.005 | 343 (10.3) | 363 (10.9) | 0.016 |
| **History of stroke /TIA** | 465 (15.1) | 447 (14.5) | 0.014 | 622 (17.1) | 642 (17.6) | 0.016 | 487 (14.7) | 491 (14.8) | 0.004 |
| **History of anaemia** | 269 (8.8) | 256 (8.3) | 0.015 | 284 (7.8) | 249 (6.8) | 0.034 | 339 (10.2) | 332 (10.0) | 0.009 |
| **History of bleeding** | 484 (15.7) | 490 (15.9) | 0.005 | 551 (15.1) | 539 (14.8) | 0.008 | 573 (17.3) | 559 (16.8) | 0.010 |
| **Use of antiplatelet drugs last 12 months** | 1 865 (60.7) | 1 871 (60.9) | 0.004 | 2 188 (60.1) | 2 209 (60.7) | 0.012 | 2 053 (61.8) | 2 055 (61.9) | 0.001 |
| **Use of NSAIDS last 12 months** | 636 (20.7) | 634 (20.6) | 0.002 | 776 (21.3) | 756 (20.8) | 0.013 | 681 (20.5) | 672 (20.2) | 0.007 |
| **Use of cholesterol lowering drugs** | 1 563 (50.8) | 1 559 (50.7) | 0.003 | 1 843 (50.7) | 1 866 (51.3) | 0.013 | 1 726 (52.0) | 1 721 (51.8) | 0.003 |
| **Mean CHA2DS2 VaSc – score (SD)** | 4.1 (1.5) | 4.1 (1.6) | 0.046 | 4.1 (1.5) | 4.1 (1.5) | 0.036 | 4.0 (1.4) | 4.0 (1.4) | 0.001 |
| **Mean HAS-BLED – score (SD)** | 2.8 (1.0) | 2.8 (1.0) | 0.022 | 2.8 (1.0) | 2.7 (1.0) | 0.004 | 2.9 (1.1) | 2.9 (1.0) | 0.006 |

Values are numbers (percent) unless otherwise specified. SD, standard deviation; SMD, absolute standardised mean difference; TIA, transient ischaemic attack; NSAIDs, non-steroidal inti-inflammatory drugs.

**Supplementary table S6. Results from sensitivity analyses. Hazard ratios of stroke/SE and major bleeding**

|  | **Main analysis^a^** | **Sensitivity analyses** | | |
| --- | --- | --- | --- | --- |
|  |  | **Follow-up restricted to**  **12 months^b^** | **Intention-to-treat analysis^c^** | **Multivariate Cox regression^d^** |
| **Dabigatran vs rivaroxaban** | | | | |
| Stroke/SE | 0.88 (0.76 - 1.02) | 0.93 (0.78 - 1.10) | 0.88 (0.78 - 0.99) | 0.92 (0.80 - 1.05) |
| Major bleeding | 0.75 (0.64 - 0.88) | 0.79 (0.64 - 0.98) | 0.84 (0.74 - 0.96) | 0.77 (0.67 - 0.90) |
| **Dabigatran vs apixaban** | | | | |
| Stroke/SE | 0.88 (0.75 - 1.02) | 0.87 (0.73 - 1.03) | 0.87 (0.78 - 1.01) | 0.91 (0.80 - 1.03) |
| Major bleeding | 1.03 (0.85 - 1.24) | 1.04 (0.82 - 1.31) | 1.06 (0.91 - 1.24) | 1.00 (0.87 - 1.16) |
| **Apixaban vs rivaroxaban** | | | | |
| Stroke | 1.00 (0.89 - 1.14) | 1.04 (0.90 - 1.20) | 0.98 (0.87 - 1.10) | 1.01 (0.90 - 1.13) |
| Major bleeding | 0.79 (0.68 - 0.91) | 0.77 (0.64 - 0.93) | 0.81 (0.71 - 0.92) | 0.77 (0.68 - 0.86) |

a. Main analysis (propensity score matching (PSM) on the basis of 16 covariates: age, gender, chronic kidney disease, hypertension, diabetes, ischaemic heart disease, peripheral artery disease (PAD), heart failure, history of stroke/SE, history of bleeding-related hospitalisation, anaemia, active cancer (cancer diagnosis last 12 months), chronic lower respiratory tract disease, use of cholesterol lowering drugs, use of antiplatelet drugs and use of NSAIDs during the last 12 months, and subsequent univariate Cox regression

b. Analysis of PSM cohorts, with follow-up time restricted to 12 months

c. Analysis of PSM cohorts, “Intention-to-treat”-like analysis where patients were not censored upon switching or discontinuation of NOACs, and were followed from index date until death or end of study period.

d. Analysis of total study population, multivariate Cox proportional hazards regression as alternative to propensity score matching (all 16 variables used in PSM).

**Supplementary figure S1. Propensity scores before and after matching**

**
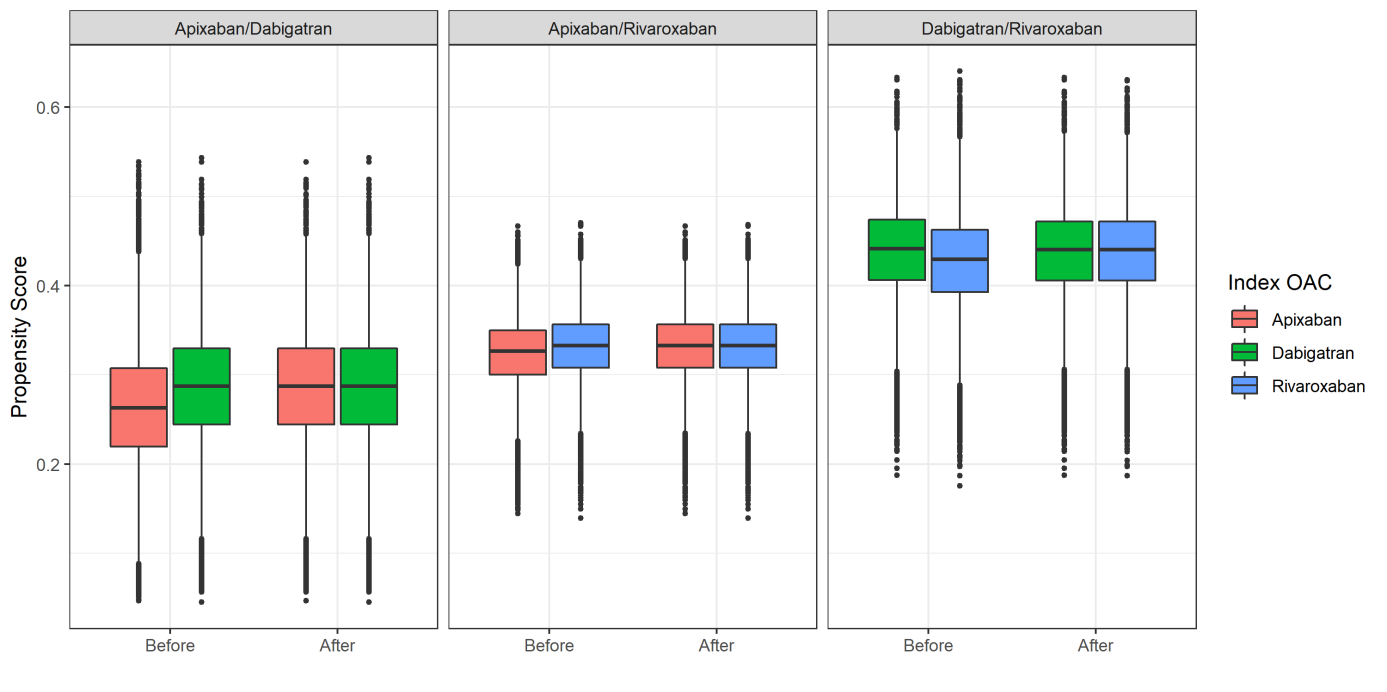
**
